# *Salmonella* *enterica* Serotype Typhimurium DT 104 Antibiotic Resistance Genomic Island I in Serotype Paratyphi B

**DOI:** 10.3201/eid0804.010375

**Published:** 2002-04

**Authors:** Danièle Meunier, David Boyd, Michael R. Mulvey, Sylvie Baucheron, Caterina Mammina, Antonino Nastasi, Elisabeth Chaslus-Dancla, Axel Cloeckaert

**Affiliations:** * Institut National de la Recherche Agronomique, Nouzilly, France; † Health Canada, Winnipeg, Manitoba, Canada; ‡ University of Palermo, Palermo, Italy; § University of Florence, Florence, Italy

**Keywords:** Salmonella genomic island I, Typhimurium DT 104, Paratyphi B

## Abstract

We have identified *Salmonella* genomic island I (SGI1) in an isolate of *Salmonella enterica* serotype Paratyphi B. This antibiotic-resistance gene cluster, which confers multidrug resistance, has been previously identified in *S. enterica* serotype Typhimurium phage types DT 104 and DT 120 and in *S. enterica* serotype Agona.

Multidrug-resistant *Salmonella enterica* serotype Typhimurium definitive phage type 104 (DT 104) has emerged during the last decade as a global health problem because of its association with animal and human disease (*1*). Multidrug-resistant strains of this phage type were first identified from exotic birds in the United Kingdom in the early 1980s and in cattle and humans in the late 1980s but have since become common in other animal species such as poultry, pigs, and sheep. The DT 104 epidemic has now spread worldwide, with several outbreaks since 1996 in the United States and Canada (*2*–*5*).

Multidrug-resistant *S.* Typhimurium DT 104 strains are commonly resistant to ampicillin, chloramphenicol/florfenicol, spectinomycin/streptomycin, sulfonamides, and tetracyclines. The antibiotic-resistance genes are clustered in part of a 43-kb genomic island called *Salmonella* genomic island I (SGI1), between the *thdf* and *int2* genes of the chromosome (*6*–*10*). The *int2* gene is part of a retron that has been detected only in serotype Typhimurium (*7*,*8*). Downstream of the retron sequence is the *yidY* gene, which is also found in the chromosome of other *S. enterica* serotypes (*7*,*8*). The antibiotic-resistance gene cluster represents approximately one third of SGI1 and is located at the 3′ end of the structure (*7*,*8*). All resistance genes are clustered and are bracketed by two integron structures (Figure 1). The first integron carries the *aadA2* gene, which confers resistance to streptomycin and spectinomycin, and a truncated *sulI* resistance gene. The second contains the beta-lactamase gene *bla*_PSE-1_ and a complete *sulI* gene. Flanked by these two integron structures are the *floR* gene (*6*), also called *floSt* (*11*) or *cmlA*-like (*9*), which confers cross-resistance to chloramphenicol and florfenicol, and the tetracycline-resistance genes *tetR* and *tet* (G). Florfenicol resistance and detection of the *floR* gene by polymerase chain reaction (PCR)-based methods have been proposed as a means for rapidly identifying multidrug-resistant *S.* Typhimurium DT 104 strains (*11*), since phage typing is available only in specialized laboratories. However, recently SGI1 has been reported in another phage type of serotype Typhimurium and in serotype Agona, suggesting horizontal transfer of SGI1 (*7*,*10*,*12*). In serotype Agona, SGI1 has the same chromosomal location as in DT 104 strains, except that it lacks the retron sequence found downstream of SGI1; thus it is located between the *thdf* gene and the *yidY* gene of the chromosome (*7*).

**Figure 1:**
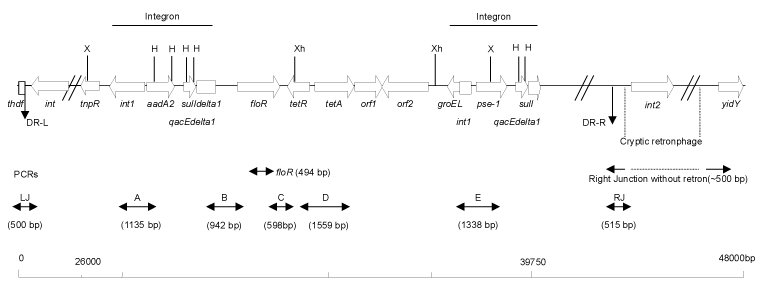
Genetic organization of the antibiotic-resistance gene cluster of SGI1 of *Salmonella enterica* serotype Typhimurium DT 104. DR-L and DR-R are the left and right direct repeats, respectively, bracketing SGI1. Polymerase chain reactions (PCRs) used to assess the genetic organization of the antibiotic-resistance genes (PCRs *floR*, A, B, C, D, and E) and the SGI1 junctions to the chromosome (PCRs LJ and RJ for left and right junctions, respectively) are indicated. Abbreviations for restriction sites: X, *Xba*I; H, *Hin*dIII; Xh, *Xho*I.

Recently, Nastasi and Mammina reported the presence of the *floR* and *tet*(G) genes detected by PCR in an *S. enterica* serotype Paratyphi B strain of biovar Java isolated from a tropical fish in Singapore (*13*). We examined this isolate to determine the presence of SGI1.

## The Study

The antibiotic-resistance phenotype of the serotype Paratyphi B strain was assessed by the disk-diffusion assay. All antibiotic disks except for florfenicol were purchased from Bio-Rad (Marnes-La-Coquette, France). Florfenicol disks and the drug itself were obtained from Schering-Plough Animal Health (Kenilworth, NJ). The serotype Paratyphi B strain showed a multidrug-resistance profile typical of serotype Typhimurium DT 104 or serotype Agona strains carrying SGI1, i.e., resistance to ampicillin, chloramphenicol and florfenicol, streptomycin and spectinomycin, sulfonamides, and tetracyclines. Moreover, this strain showed the same resistance level to florfenicol as serotypes Typhimurium DT 104 and Agona, i.e., a florfenicol MIC 32 µg/mL (*12*). The strain was susceptible to trimethoprim and the quinolones nalidixic acid, enrofloxacin, and ciprofloxacin.

No plasmids were detected in the serotype Paratyphi B strain, suggesting that all antibiotic-resistance genes were chromosomally located. PCR mapping of the typical antibiotic resistance genes and integrons associated with SGI1 was performed as described (Figure 1) (*12*). PCR amplifications yielded products from genomic DNA extracted from the serotype Paratyphi B strain of the size expected from DNA of serotype Typhimurium DT 104 control strain BN9181 (data not shown) (*6*,*12*). Partial nucleotide sequencing of the *floR* gene showed 100% identity with that of serotype Typhimurium DT 104. Thus, these PCR mapping results indicated that the serotype Paratyphi B strain contains the entire antibiotic-resistance gene cluster of serotype Typhimurium DT 104.

**Figure 2:**
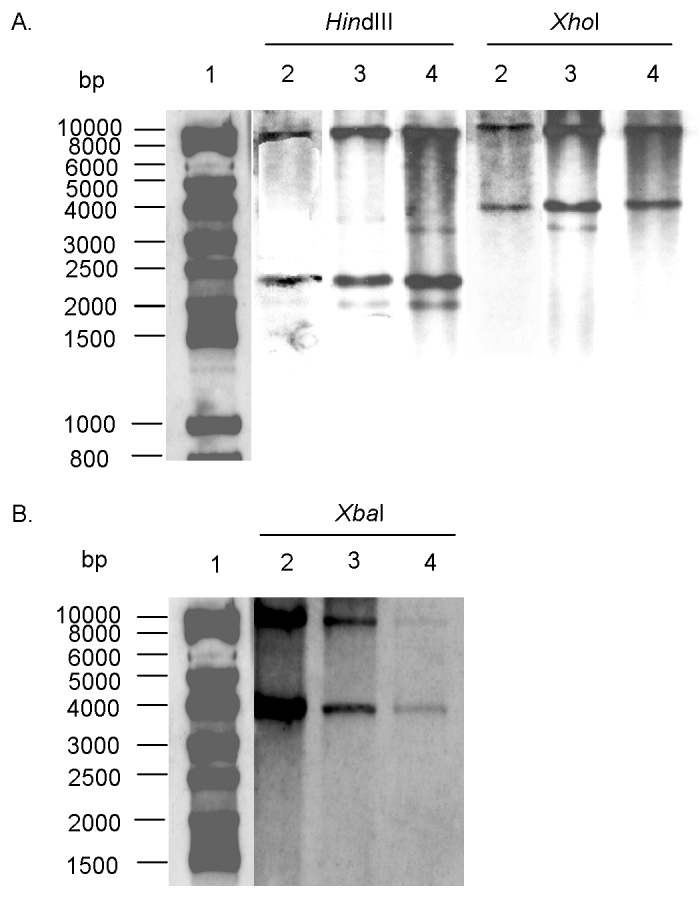
**A.** Southern blot hybridization with the *Xba*I probe (Figure 1) containing all antibiotic-resistance genes of *Hin*dIII- and *Xho*I-digested genomic DNAs of *Salmonella enterica* serotype Typhimurium DT 104 strain BN9181 (lanes 2), serotype Agona strain 959SA97 (lanes 3), and serotype Paratyphi B strain (lanes 4). Lane 1: DNA ladder. **B.** Southern blot hybridization with the p1-9 probe of *Xba*I-digested genomic DNAs of *S. enterica* serotype Typhimurium DT 104 strain BN9181 (lanes 2), serotype Agona strain 959SA97 (lanes 3), and serotype Paratyphi B strain (lanes 4). Lane 1: DNA ladder.

The conservation of the antibiotic-resistance gene’s organization was further assessed by Southern blot of *Hin*dIII- or *Xho*I-digested genomic DNA, with the 12-kb *Xba*I insert as probe (see *Xba*I fragment, Figure 1) from recombinant plasmid pSTF3 containing the DT 104 antibiotic-resistance gene cluster as described (*6*,*12*). The Southern blot profiles of the serotype Paratyphi B strain were identical to those of the DT 104 control strain BN9181 and serotype Agona strain 959SA97 (Figure 2), confirming that the organization of the antibiotic-resistance genes in the serotype Paratyphi B strain is the same as in serotype Typhimurium DT 104 or serotype Agona containing SGI1 (*7*,*12*).

To assess the presence of entire the SGI1 and its location in the chromosome of the serotype Paratyphi B strain, PCR was performed by using primers corresponding to the left and right junctions of SGI1 in the *Salmonella* chromosome. PCR took into account the presence or absence of the *int2*-retron sequence, which is located downstream of SGI1 in serotype Typhimurium DT 104 but not in serotype Agona (Figure 1) (*7*,*8*). PCR was positive for the left junction of SGI1, as for serotype Typhimurium DT 104 or serotype Agona. If a sequence of the *int2* gene of the retron was used as reverse primer, PCR was negative for the right junction of SGI1, but it was positive if the sequence of the *yidY* gene was used. The PCR products showed the expected sizes of approximately 500 bp for both the left junction and right junction PCR without the retron sequence in Figure 1. These data indicate that the serotype Paratyphi B strain contains SGI1 at the same chromosomal location as in serotype Typhimurium DT 104 or serotype Agona, that is, between the *thdf* and *yidY* genes, but lacks the retron sequence found in DT 104 strains and other serotype Typhimurium strains (*7*,*8*). The presence of entire SGI1 was also confirmed by Southern blot of *Xba*I-digested genomic DNA with the p1-9 probe containing a 2-kb *Eco*RI insert. This corresponds to a central region of SGI1, comprising parts of the S023 and S024 open reading frames, which code for putative helicase and exonuclease proteins, upstream of the antibiotic-resistance gene cluster (*7*). This probe revealed *Xba*I fragments of the 4- and 9-kb sizes expected in the serotype Paratyphi B strain and the control serotype Typhimurium DT 104 and serotype Agona strains (Figure 2B).

**Figure 3:**
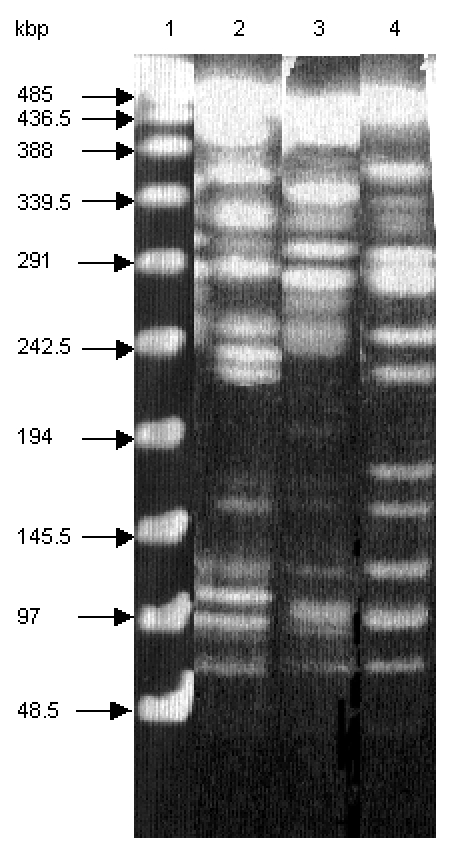
Macrorestriction analysis by pulsed-field gel electrophoresis of genomic DNAs cut by *Xba*I of *S. enterica* serotype Typhimurium DT 104 strain BN9181 (lane 2), serotype Agona strain 959SA97 (lane 3), and the serotype Paratyphi B strain (lane 4). Lane 1: DNA ladder.

Macrorestriction analysis by pulsed-field gel electrophoresis of the serotype Paratyphi B strain DNA cut by *Xba*I showed that it is genetically distinct from both serotypes Typhimurium DT 104 and Agona (Figure 3), further indicating at the molecular level that the occurrence of SGI1 in the serotype Paratyphi B strain probably results from horizontal transfer and not seroconversion of known *S. enterica* serotypes containing SGI1.

## Conclusions

We have identified SGI1 in a *S.* Paratyphi B strain. These data, in conjunction with the identification of SGI1 in *S.* Agona and *S.* Typhimurium strains, suggest horizontal transfer of this region (*7*,*10*,*12*). That SGI1 has the same chromosomal location in *S.* Typhimurium, *S.* Agona, and *S.* Paratyphi B suggests that its insertion occurred through a homologous recombination event, perhaps through phage transduction (*14*). This hypothesis is experimentally supported by the fact that resistance genes of serotype Typhimurium DT 104 can be efficiently transduced by P22-like phage ES18 and phage PDT17, which is released by all DT 104 isolates analyzed (*14*). However, the question remains why the retron sequence downstream of SGI1 in serotype Typhimurium DT 104 and DT 120 strains (*7*) is not present downstream of SGI1 in other serotypes. A possible explanation could be that in the horizontal transfers described here, the SGI1 donor strains are not serotype Typhimurium strains. Once SGI1 has been acquired, it may become stable in the chromosome, as in vitro excision experiments failed to demonstrate its loss in a DT 104 strain in the absence of antibiotic selective pressure (*8*). This factor may contribute to evolution of *S. enterica* pathogens, similar to the acquisition of pathogenicity islands (*15*,*16*).

Acquisition of SGI1 may have been a key factor contributing to the DT 104 worldwide epidemic, perhaps not only through selection by agricultural use of antimicrobial agents (*17*) but also by virulence properties of SGI1 (*10*). Therefore, further surveillance is warranted for the emergence of horizontal transfer of SGI to *S. enterica* serotypes of public health importance.
